# Highly Stretchable and Conductive Silver Nanoparticle Embedded Graphene Flake Electrode Prepared by *In situ* Dual Reduction Reaction

**DOI:** 10.1038/srep14177

**Published:** 2015-09-18

**Authors:** Yeoheung Yoon, Khokan Samanta, Hanleem Lee, Keunsik Lee, Anand P. Tiwari, JiHun Lee, Junghee Yang, Hyoyoung Lee

**Affiliations:** 1 Center for Integrated Nanostructure Physics, Institute for Basic Science (IBS), Sungkyunkwan University, Suwon 440-746. Korea; 2 Department of Chemistry, Department of Energy Science, SKKU Advanced Institute of Nano Technology (SAINT), Sungkyunkwan University, Suwon 440-746. Korea; 3 Samsung-SKKU Graphene Center (SSGC), Sungkyunkwan University, 2066 Seoburo, Jangan-Gu, Suwon, Gyeonggi-Do 440-746, Republic of Korea

## Abstract

The emergence of stretchable devices that combine with conductive properties offers new exciting opportunities for wearable applications. Here, a novel, convenient and inexpensive solution process was demonstrated to prepare *in situ* silver (Ag) or platinum (Pt) nanoparticles (NPs)-embedded rGO hybrid materials using formic acid duality in the presence of AgNO_3_ or H_2_PtCl_6_ at low temperature. The reduction duality of the formic acid can convert graphene oxide (GO) to rGO and simultaneously deposit the positively charged metal ion to metal NP on rGO while the formic acid itself is converted to a CO_2_ evolving gas that is eco-friendly. The AgNP-embedded rGO hybrid electrode on an elastomeric substrate exhibited superior stretchable properties including a maximum conductivity of 3012 S cm^-1^ (at 0 % strain) and 322.8 S cm^-1^ (at 35 % strain). Its fabrication process using a printing method is scalable. Surprisingly, the electrode can survive even in continuous stretching cycles.

Stretchable electronics has been perceived as an alternative technology for the realization of the next generation wearable electronic devices. Stretchable electronic circuits and electrodes would enable expandable, foldable and adhesive electronic devices for wearable or epidermal electronic devices. For the fabrication of stretchable electrodes, until now, an integration of carbon based materials, especially 1D carbon nanotubes (CNTs), with rigid inorganic conductive materials has been used. The rigid and brittle inorganic conductive materials are embedded in chemically and/or physically bonded onto the surface of carbon nanotubes^1^. The resulting embedded inorganic materials to the carbon nanotube electrodes show high conductivity and stretchability. Although the inorganic materials-embedded carbon nanotubes that are one of the various carbon allotropes showed possibility as a stretchable electrode with high conductivity, there are still unsolved big obstacles in the realization of the stretchable electrodes, especially for a poor device fabrication process such as poor solubility in common solvents and high fabrication cost by using expensive raw materials. Due to the poor solubility of inorganic materials and carbon nanotubes in water and other organic solvents, it is hard to prepare the homogeneous inorganic materials/carbon nanotube composites with a simple reaction under solution process.

On the contrary, graphene oxide (GO) derived from graphite with acid treatments, a single layer of sp^2^ and sp^3^-bonded carbon atoms arrayed into a paradigmatic 2D carbonaceous nanomaterial, has attracted wide interest due to its extraordinary solubility and easily achievable chemical functionalities with other inorganic materials under various solution process. In addition to GO, reduced GO (rGO) have attracted much attention in various research fields such as sensing^2^,^3^,^4^,^5^,^6^,^7^,^8^, nanoelectronics^9^,^10^,^11^,^12^, energy-storage^13^,^14^,^15^, catalysis^16^, and nanobiotechnology^17^,^18^. Recently, metal fabricated-rGO/hybrid materials or nanocomposites have attracted intense research interest due to their optical, electronic, thermal, mechanical, and catalytic properties^19^,^20^,^21^,^22^,^23^. An overall goal is to fabricate composites or hybrid materials which can integrate GO or rGO with polymers, metal nanoparticles (NPs) or even nanotubes and fullerenes. Due to a large surface area and the aforementioned properties, GO or rGO has been an attractive alternative choice as the matrix for nanocomposites.

Until now, however, few works have reported the use of GO or rGO as a template to directly synthesize metal NPs and directly fabricate metal NP-GO composites on substrates. Metal NPs are of great importance due to their optical, catalytic, electrical and antimicrobial properties^24^. The fabrication of metal NPs into composite materials is also of interest to explore their properties and applications. Therefore, integration of metal NPs with GO or rGO and the synthesis of metal nanoparticles using GO or rGO as a template are key research interests. Muszynski *et al.* have synthesized Au NPs using the chemical reduction of HAuCl_4_ with NaBH_4_^25^. In this method, they used a graphene-octadecylamine suspension in THF and used metal borohydride as a reducing agent. However, the process is not eco-friendly. Furthermore, the gold particles were anchored on the octadecylamine functionalized graphene, not directly in the graphene surface. Recently, Nanda *et al.* synthesized M@rGO using zinc and H_2_SO_4_^26^. They used Zn-acid medium for the production of another metal NP such as Au, Pt, Pd and Ag. Highly concentrated H_2_SO_4_ (10 M) was required for the production of small metal NPs, lower concentrations resulted in large NPs (50 nm). However, these methods produced unsuitable materials for the use as conductive materials in the stretchable conducting electrodes. Thus, even though there are many publications on the preparation of stretchable conductive electrodes using AgNWs^27^, fibres^28^ and carbon nanotubes^1^, however, there is no report to use rGO-metal NPs hybrid composites. It is stongly suggested that if we carefully prepare homogeous AgNP-embedded rGO film on the elastomeric polymers, the rGO-AgNP film through a printing process that is compatible with any substrate is expected to be a highly conductive and also highly stretchable electrode, which is scalable for the printable electronic devices.

Herein, we report a novel method to prepare new hybrid rGO-AgNP conducting materials by carefully designed reduction duality of formic acid at low temperature (Fig. 1a). To get the homogenous rGO-metal NP film without any kinds of aggregation, a dispersibility issue in any solvent system is very important. Since a conductive rGO and silver NP are not dispersible in water at all, to find an *in-situ* direct reduction of dispersible GO to rGO and simultaneously direct reduction to dispersible Ag ion into Ag metal NP in aqueous phase is a key issue. Fortunately, we found that the formic acid could reduce silver positive ion into silver metal NP and also simultaneously reduce GO into rGO in aqueous phase. As expected, these composite materials can be applied for highly stretchable conductive electrodes using a simple printing technique. And we also like to report how the as-prepared hybrid rGO-AgNP conducting electrode on a polymer matrix can be stretched out, compared to the commercially available Silver paste electrode on the same polymer matrix.

## Results

### *In-situ* synthesis of metal embedded graphene materials by reduction duality of formic acid

We carefully designed an *in situ* synthesis of rGO-metal NPs by using reduction duality of the formic acid in presence of AgNO_3_ or other metal salts. Here, AgNO_3_ acts as an initiator of the reduction process as well as the source of AgNPs. Once the reduction process starts, the positively charged novel metal (Ag and Pt) can make formic acid into carbon dioxide, two protons, and electrons which were generated in reaction solution. This step is very critical to determine the reaction rate in whole reduction process. Although the protons could diffuse away into the solution, the generated electrons would transfer to positively charged novel metal to reduce them into metal NPs. Then the excess electrons would accumulate on the surface of metal NPs. When the metal NPs attach onto the GO sheets, the electrons would flow to the GO sheets and the oxygen functional groups from the GO sheets in the water media with aid of protons would be reduced. In presence of formic acid, the high concentration of the protons would inhibit the rate of the reaction. Thus, the reduction duality of the formic acid can convert graphene oxide (GO) to rGO and also simultaneously deposit the positively charged metal ion to metal NP on the rGO nanosheets. We have postulated mechanism for the reduction duality of a formic acid, indicating the conversion of metal ions to metal nanoparticles and also simultaneous reduction of GO into rGO.


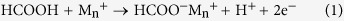



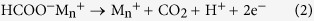



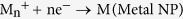



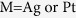



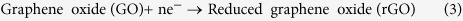


**Figure 1:**
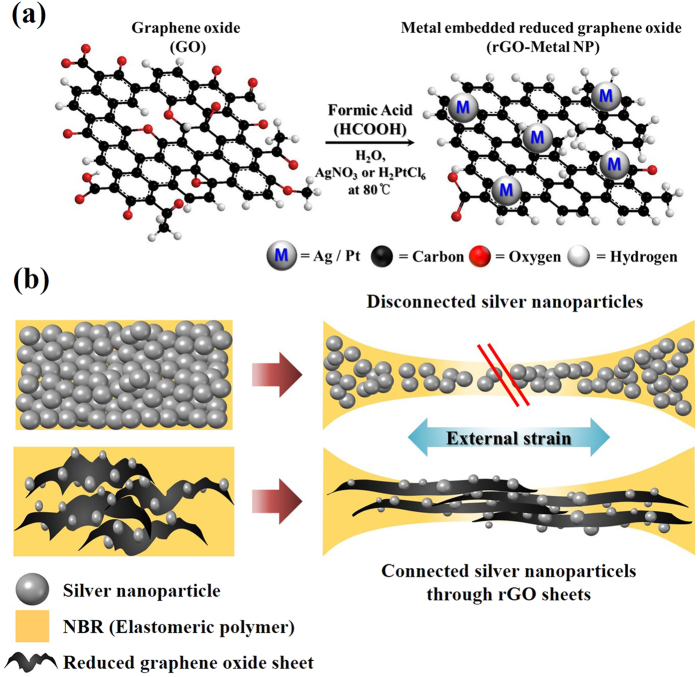
Schematic illustration of *in situ* synthesis of metal nanoparticle embedded reduced graphene oxide (rGO-AgNP) and possible stertchable mechanism of rGO-AgNP hybrid films in a polymer matrix. (**a**), Synthesis of metal-embedded rGO from GO by reduction duality of formic acid. (**b**), a schematic representation of the stretchable and conductive rGO-AgNP hybrid films.

In a typical reaction, 40 mg GO was dispersed in DI water, and then 1–2 mL of formic acid and a catalytic amount (5 mg) of AgNO_3_ were added. The smallest amount of AgNO3 is 3 mg to reduce GO and metal precursor into rGO-metal NPs). The reaction mixture was then heated to 80 °C to yield rGO-AgNP hybrid materials (Fig. 1a). We also carried out these reactions taking formic acid and AgNO_3_ separately under similar conditions for the control experiments. GO was not reduced, and no AgNPs were generated in either case. When catalytic amounts of AgNO_3_ were added to the formic acid, the reaction performed well. As we mentioned earlier, AgNO_3_ initiated the reduction of GO by formic acid, and once the reduction process began, formic acid reduced both the GO and AgNO_3_ simultaneously to produce AgNP-embedded rGO hybrid materials *in situ*. The formic acid plays a dual role to reduce GO into rGO and simultaneously convert Ag ions to AgNP via a reduction process^29^.

During the reaction process, slow evolution of a gas with bubble formation was observed, which is a good agreement with our proposed mechanism where CO_2_ gas was produced during the formic acid reduction process^29^. In this regard, different amount of AgNP-fabricated rGOs from 0.6 wt% to 20.01 wt% of AgNP were prepared by varying AgNO_3_ concentration. This method was also successfully extended to prepare PtNP-embedded rGO (rGO-PtNP) in a similar manner.

### Reduction degree of GO/AgNO_3_ to rGO-AgNP depends on amount of AgNO_3_

**Figure 2:**
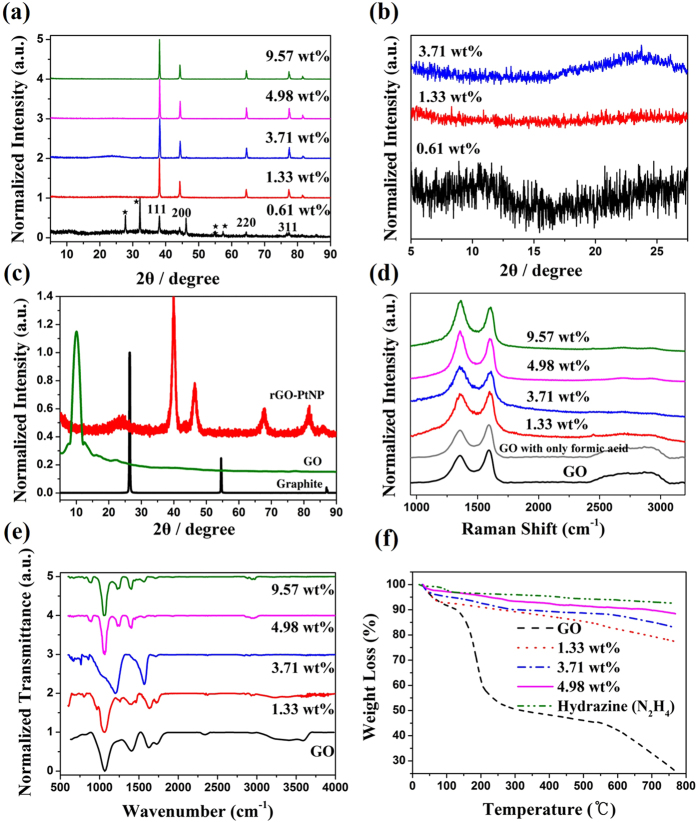
Compositional analysis of the prepared hybrid materials. (**a**), XRD spectroscopy of amount of AgNP dependent rGO-AgNP materials. (**b**), Partial amplified XRD spectroscopy of rGO-AgNP hybrid materials (0.61, 1.33 and 3.71 wt%), indicating the AgNO_3_ act as a initiator to reduce the GO. (**c**), XRD spectra of rGO-PtNP synthesized using formic acid and hexachloroplatinic acid (H_2_PtCl_6_). (**d**,**e**), Raman and FT-IR spectroscopy of GO and the amount of AgNP dependent rGO-AgNP. (**f**) Thermogravimetric analysis of GO and rGO-AgNP hybrid materials (1.33, 3.71 and 4.98 wt%).

Ag and Pt on rGO were characterized by X-ray diffraction (XRD). Figure 2(a–c) showed the XRD pattern of the amount of AgNP dependent rGO-AgNP and rGO-PtNP, respectively (Fig. 2a,c), along with GO and graphite. The 2*θ* peak of graphite powder was found at 26.71^°^, indicating that the interlayer distance was 3.34 Å (Fig. 2c). The 2*θ* peak of GO appeared at 10.27 ^°^, corresponding to an interlayer distance of 8.60 Å (Fig. 2c). Peaks at 38.1^°^, 44.3^°^, 64.5^°^ and 77.3^°^ correspond to the strongest reflections of (1 1 1), (2 0 0), (2 2 0) and (3 1 1) crystallographic planes of the Ag NP phase, respectively (Fig. 2a)^30^. The peaks at 39.9^°^, 46.6^°^, 68.1^°^, and 81.7^°^ correspond to the strongest reflections of (1 1 1), (2 0 0), (2 2 0) and (3 1 1) crystallographic planes of the Pt NP phase, respectively (Fig. 2c)^31^. Amazingly, Fig. 2b is a magnified results from diffraction peaks of GO and rGO in Fig. 2a, indicating that GO was beginning to reduce as more AgNO_3_ was added and finally the diffraction peak of ~10^°^ was shifted to ~24^°^. This phenomenon in XRD is well-known as the reduction process in GO due to the removal of oxygen groups^32^. In addition, it means that AgNO_3_ can act as an initiator to reduce GO as mentioned earlier. The peak at 24.1^°^ belongs to the (002) reflection of rGO^33^. Raman spectroscopy was used to further examine the effect of fabrication of metal nanoparticles and simultaneous reduction of GO with this reaction. Figure 2d shows the Raman spectra of the GO powder and amount of AgNP dependent rGO-AgNP hybrid materials, and Fig S1a shows the Raman spectra of GO and rGO-PtNP. The Raman spectra of GO and metal nanoparticle-embedded rGO as prepared in this method were all similar. All spectra showed a D-band (due to defect), G-band (originating from in-plane optical vibrations), and 2D band (from a two phonon scattering process), and a peak near 2980 cm^−1^ (S3-band)^34^. However, when the amount of AgNP by adding more AgNO_3_ was increased, the *I*_D_/*I*_G_ ratio changed significantly from GO to AgNP of 9.57 wt%. The *I*_D_/*I*_G_ ratio of GO, AgNP of 1.33, 3.71, 4.98 and 9.57 wt% on rGO were 0.71, 0.98 1.12, 1.18 and 1.21, respectively, which increased from 0.71 (GO) to 1.21 (9.57 wt%). This was because the functionalized sp^3^ hybridized C–C bonds in the GO transform to sp^2^ hybrid C–C double bonds in rGO, which increases disorder in the basal plane of rGO; the higher de-oxygenation of GO implies a high quality reduction of GO to rGO. Furthermore, the enhanced intensity of 2D and S3 peaks at around 2690 cm^-1^ and 2950 cm^-1^ for the rGO (Fig. 2d) are attributed to the good reduction of GO. Additionally, to determine the effect of AgNO_3_ as an initiator in this reduction reaction, we carried out Raman measurement for GO with only formic acid. As we expected above, it was not reduced in the absence of AgNO_3_ which is in good agreement with our postulated mechanism (Fig. 2d). Fourier transform infrared spectroscopy (FT-IR) was further used to investigate the reduction process. Figure 2e shows the FT-IR spectra of GO and different amount of AgNP of the rGO-AgNP hybrid materials. The characteristic peaks in the IR spectra of GO (Fig. 2e) were ~3409 cm^-1^ (broad, O–H stretching), 2948 cm^-1^ (CH_2_ stretching), 1728 cm^-1^ (C = O stretching), 1632 cm^-1^ (C = C stretching) and 1404 cm^-1^ (O–H bending)^35^,^36^. From the IR spectra, it is clear that all rGO-AgNP hybrid materials showed no peak at 1404 or 3409 cm^-1^ and a very low intensity at 1728 cm^-1^, indicating the removal of the maximum number of oxygenated groups present in GO. IR spectra of rGO-PtNP are given in Fig S1b and provide good evidence for *in-situ* reduction during metal fabrication on GO. Thermogravimetric analysis (TGA) was used to further assess the level of the reduction of the rGO-AgNP hybrid materials. Figure 2f displays the TGA thermograms that show weigh loss as a function of temperature for GO and different amount of AgNP of the rGO-AgNP hybrid materials under N_2_ atmosphere. The GO showed significant weight loss with an onset temperature at slightly more than 100 °C, which was attributed to the elimination of interlamellar water^33^, followed by loss of oxygen of the GO themselves at slightly higher temperatures. Interestingly, the TGA stability of the rGO-AgNP hybrid materials has sharply improved as the amount of the AgNO_3_ initiator was increased because of the better graphitization and de-oxygenation of the rGO-AgNP hybrid materials with enhanced van der Waals force interaction between layers^33^.

**Figure 3:**
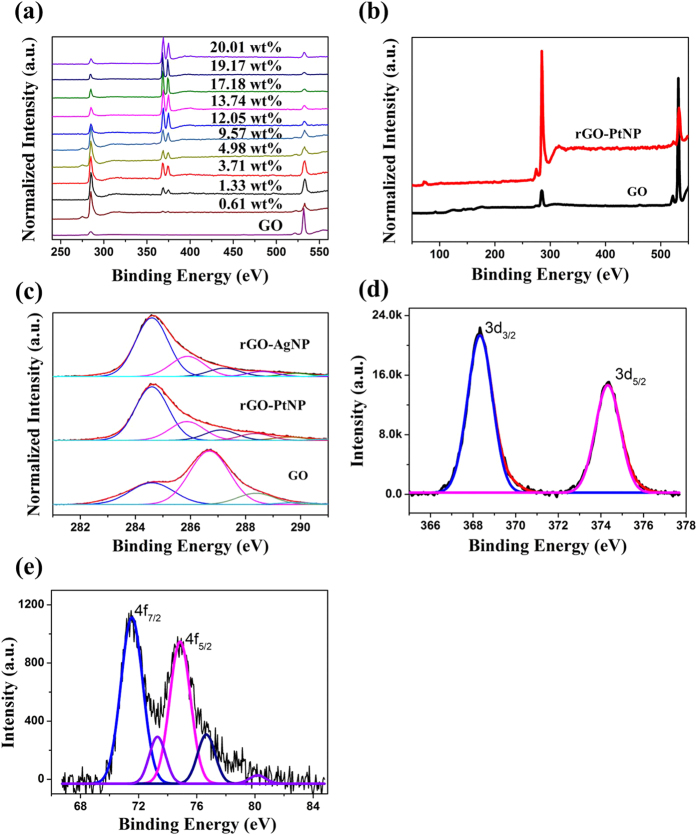
Compositional analysis. (**a**), Survey results in XPS spectroscopy of GO and different Ag nanoparticle-embedded rGOs prepared using formic acid and AgNO_3_ from GO. (**b**), Survey results in XPS spectroscopy of GO and Pt nanoparticle-embedded rGO prepared by using formic acid and H_2_PtCl_6_ from GO. (**c**), C1s results in XPS spectroscopy of GO, rGO-AgNP and rGO-PtNP. (**d,e**), Ag 3d and Pt 4f results in XPS spectroscopy of rGO-Ag and PtNP.

Incorporation of metal nanoparticles in rGO was also characterized using X-ray photoelectron spectroscopy (XPS). Figure 3a shows a comparison of the XPS spectra of GO and varying atomic weight percentage (0.61, 1.33, 3.71, 4.98, 9.57, 12.05, 13.74, 17.18, 19.17 and 20.01 wt%) of AgNP in the rGO-AgNP hybrid materials, while Fig. 3b shows the difference between GO and rGO-PtNP. Figure 3c shows typical C1s spectra in XPS results for GO, rGO-AgNP (1.33 wt% of AgNP) and rGO-PtNP, and Fig. 3d and e show Ag 3d XPS spectra of rGO-AgNP and Pt 4f XPS spectra of rGO-PtNP. Fig S2a and S2b show high resolution C1s XPS spectra of various rGO-AgNPs having different amount of AgNPs, and their Ag 3d XPS spectra, respectively. An obvious Ag 3d doublet peak arose at 368.76 eV (Ag 3d_5/2_) and 374.79 eV (Ag 3d_3/2_), confirming the formation of AgNPs in rGO (Fig. 3d and S2b)^37^. The doublet peaks of Ag 3d arose from spin–orbit coupling (3d5/2 and 3d3/2)^37^. As an amount of AgNPs on rGO-AgNP hybrid materials increased, the oxygen functional groups were drastically decreased from 0.61 wt% of AgNPs and gradually decreased as amount of AgNPs increased to 20.01 wt% (Fig. 3c and S2), indicating the effectiveness of the reduction. Interestingly, when we added smaller amount of AgNO_3_ into reaction solution than that of 0.61 wt%, both rGO and AgNPs are not formed in our experimental system. Thus, the generation of protons and electrons in reaction solution is very critical to determine the reaction rate in whole reduction process.

**Figure 4:**
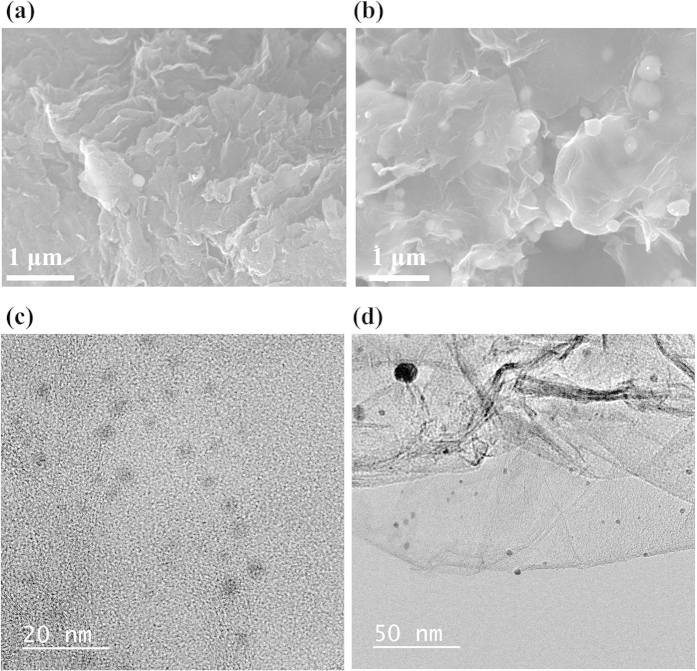
Scanning Electron Microscopy (SEM) and transmission electron microscopy (TEM) of the morphology and microstructure in rGO-AgNP and rGO-PtNP hybrid materials. (**a**) and (**b**), SEM images of rGO-AgNP and rGO-PtNP, repectively. (**c,d**) TEM images of rGO-AgNP and rGO-PtNP, repectively.

Scanning electron microscopy (SEM) and transmission electron microscopy (TEM) were also employed to demonstrate the decoration of AgNPs on rGO and to calculate the size of the nanoparticles embedded on rGO. Figure 4a and b show SEM images of rGO-AgNP and rGO-PtNP, and Fig. 4c,d and S3 show TEM images of rGO-AgNP and rGO-PtNP. It is evident from the TEM images that the size of the particles is around 5–15 nm. AFM images shown in Fig S4 also showed the uniform size distribution of the metal nanoparticles. The SEM morphology of our prepared compounds as shown in Fig. 4a,b and S5 clearly indicates that the metal nano particles were deposited and embedded on rGO during the reaction condition. Fig S4 shows the SEM images of rGO-AgNP hybrid materials having various amount of AgNPs, indicating that morphologies of AgNP on rGO were gradually aggregated as the amount of AgNP increased due to the surfactant-free reaction. This is also good evidence for the formation of a nanoparticle-rGO hybrid structure.

### Fabrication and characterization of rGO-AgNP based stretchable hybrid films

To prepare stretchable and conductive rGO-AgNP hybrid films, as-prepared rGO-AgNP powder was grounded and sonicated in a polyvinylidenefluoride (PVDF) solution. PVDF copolymer was chosen as a matrix because of its good electrical and mechanical properties^1^,^38^. Firstly, the rGO-AgNP hybrid film with an average thickness of 30 μm on PET or glass substrate was prepared by doctor blade technique, and then for an embedding, elastomeric polymer solution such as nitrile butadiene rubber (NBR) was poured onto the rGO-AgNP film. Finally, the rGO-AgNP hybrid film was easily peeled off from the substrate after drying and curing with hot-roll pressing at 150 °C (Fig S6). The conductivity of the rGO-AgNP hybrid film was investigated as a function of a curing temperature (Fig S7). The conductivity increased with the increased curing temperature because of a shrinkage of the polymer matrix^1^.

**Figure 5:**
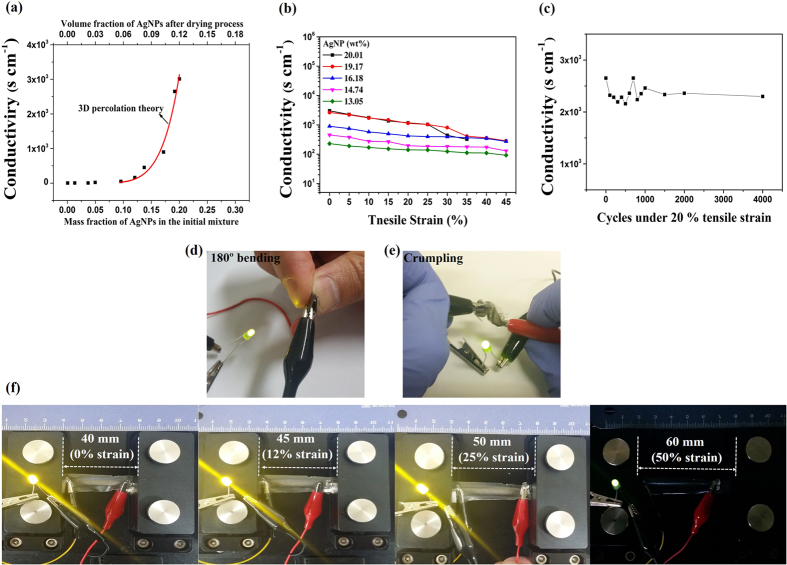
Electrical characteristics of rGO-AgNP hybrid films. (**a**), Conductivity of the hybrid rGO-AgNP film, composed of rGO decorated with varing amount of silver nanoparticles, invetigated at 0 % strain. The red line is a prediction based on a power-law realationship and three dimensional percolation theory. (**b**), Conductivity of the hybrid rGO-AgNP films under tensile strain for five different fraction of silver nanoparticles. (**c**), cycling test of rGO-AgNP hybrid film (19.17 wt%) under 20% tensile strain. (**d,e**), Operation of the LED chips connected to the hybrid rGO-AgNP hybrid film (19.17 wt%) and visual images of the LEDs at an applied viltage of 3.0 V after 180° bending and crumpling. (**f**) Before (0 % strain) and after stretching (12%, 25 % and 50 % strain, respectively).

Figure 5a shows the conductivity of the rGO-AgNP hybrid films at 0 % strain as a function of a mass faction of AgNPs. The masses of the other components (rGO 100 mg, 10 wt% of PVDF solution in NMP 100 μL) were fixed. The conductivity of the rGO-AgNP hybrid film was started to increase when the mass fraction of AgNPs was reached at a 9.0 wt%. The mass fraction of AgNP above 20.01 wt% resulted in a brittle film with a phase separation. Surprisingly, a bare AgNP film was not stretchable at all and had a relatively low conductivity. Theoretical prediction for the conductivity of the hybrid film was calculated using a power-law relationship and 3D percolation theory (see Supporting Information). Simply, the power-law relationship^39^ is describe by


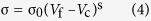


where σ is the electrical conductivity of the composite, σ_0_ is the conductivity of the conductive filler, *V*_f_ is the volumetric fraction of the filler, *V*_c_ is the volumetric fraction at the percolation threshold, and *s* is the fitting exponent. AgNPs were modeled as uniformly distributed nanoparticles with a random orientation, and the percolation threshold was calculated using the average interparticle distance model^28^,^39^. The calculated percolation threshold (5.86 vol% after the drying process, which is equivalent to 9.57 wt% in the initial mixture) and the power-law relationship indicated good agreement with our experimental results. Figure 5b shows that the conductivity of the rGO-AgNP hybrid film (40 × 5 × 0.03 mm) was measured under varying tensile strains using four-point probe system. The as-prepared hybrid film embedded in elastomeric polymer showed a maximum conductivity around 3012 S cm^-1^ at 0% strain and the conductivity of the rGO-AgNP hybrid film was reduced with an increasing strain, and a conductivity of 322.8 Scm^-1^ was measured at 35% strain. The mass fraction of AgNPs rarely affected a stretchability, and all hybrid films were ruptured at 50 % strain. Cycling test was carried out up to 4,000 cycles, which are shown in Fig. 5c. Conductivity was initially fluctuated and then stabilized after 1,500 cycles. The electrical performance of the rGO-AgNP hybrid film with a varying strain was visually determined using green LED chips. The visual images of the LED chips before (0 % strain) and after stretching (12 %, 25% and 50% strain) are shown in Fig. 5f. The LED was turned on when the applied bias was around 3.0 V due to the energy bandgap of the LED. The brightness of LED chip was reduced when the film was stretched as increasing tensile strain (up to 50 %), indicating that resistance was increased as tensile strain increased. Those results are matched with Fig. 5b. In addition, the rGO-AgNP hybrid film was maintained their performance after 180º bending and crumpling test (Fig. 5d–e and Fig S8).

**Figure 6:**
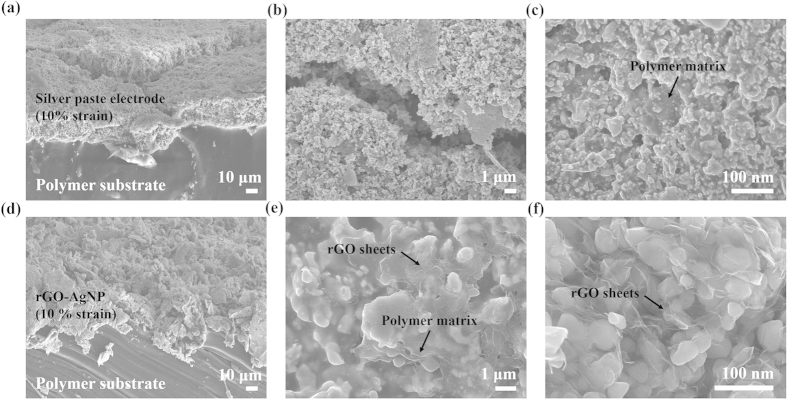
SEM images of silver paste electrode and rGO-AgNP hybrid films on a elastomeric substrate under 10 % strain. (**a–c**) SEM images of silver paste electrode on a elastomeric substrate under 10 % strain. (**d–f**) SEM images of rGO-AgNP hybrid films on a same elastomeric polymer under 10 % strain. (a and d) cross sectional images and (**b–c** and **e–f**) indicated top view images of silver paste electrodes and rGO-AgNP hybrid films, repectively.

To explain possible mechanism for stretchability of the rGO-AgNP hybrid films, we carried out Scanning Electron Microscopy (SEM) for imaging their morphologies under 10 % strain. From the SEM images, we can compare with the morphologies of the silver paste electrodes without rGO. Figure 6a,d show cross sectional SEM images of silver paste electrodes and AgNP embedded rGO on a elastomeric polymer substrate after 10 % strain, respectively. Although surface morphologies of rGO-AgNP hybrid films are rough more than those of silver paste electrode without rGO, there is big cracks in case of only silver paste electrodes in magnified images (Fig. 6b). This phenomenon may explain from Fig. 6c,f. The silver paste without rGO shown in Fig. 6c exhibits that the Ag particles are segregated from the non-conductive matrix, elastomeric polymer, NBR. On the contrary, the soft and flexible rGO sheets with high aspect ratios may construct effective electrical networks between AgNPs, and the AgNPs adsorbed on the surface of the rGO sheets may enhance the contact interface (Fig. 1b and Fig. 6f). The simplest explanation is that although silver paste electrodes shows dense morphologies with polymer matrix, they are still rigid and brittle under external strain. In contrast, the rGO-AgNP hybrid film clearly shows the formation of the electrically contacted networks even under strain, leading to give a high strechability.

## Discussion

In conclusion, a novel, convenient, and inexpensive solution process were demonstrated for fabrication of metal NP-embedded rGO hybrid materials using reduction duality of formic acid. The highly conductive and stretchable electrode with a high conductivity 3012 S cm^-1^ at 0 % and 322.8 S cm^-1^ at 35 % strain was successfully prepared using printable metal embedded rGO ink on a substrate that provided highly stretchable through elastomeric polymer embedding process. According to the power-law relationship of equation (1), this can be minimized or overcome by using a polymer matrix with a poisson’s ratio, because the in *V*_f_ or increase in total volume can be reduced. A composite with an excessive amount of silver may exhibit long-term instability, because phase separation of AgNP was observed when the concentration was higher than 20.01 %. These wet-processible, stable rGO-metal hybrid materials can be applied to a graphene based conductive ink for the stretchable electrode including large-area electronic circuit, epidermal electronics and wearable energy storage devices as charge collectors, and in other modern nanoelectronics.

## Method

### Materials

Natural graphite (Bay Carbon, SP-1 graphite), sulfuric acid (95–97%), hydrogen peroxide (30 wt.%), potassium permanganate, sodium nitrate, silver nitrate and formic acid were obtained from commercial sources and used as received.

#### Preparation of Graphene Oxide (GO)

Graphene oxide (GO) was prepared from natural graphite powder by the modified Hummers and Offenman’s method using sulfuric acid, potassium permanganate, and sodium nitrate^33^.

### Preparation of rGO-metal nanoparticle hybrid materials (rGO-AgNP and rGO-PtNP)

GO was dispersed in DI water (40 mL, 2 mg mL^-1^) and then 1 ~ 2 mL of formic acid and a catalytic amount (5 mg) of AgNO_3_ were added to it (to prepare the AgNP of 1.33 wt% in rGO-AgNP hybrid material). The reaction mixture was then heated to 80 °C with stirring for 6 hours. It was then filtered and first washed with deionised water for several times and then with saturated sodium bicarbonate solution to wash away excess formic acid and washed again with deionised water for several times. It was then dried under vaccum at 60 °C for 24 hours to yield the rGO-AgNP hybrid materials. Varying the amount of AgNO_3_, different rGO-AgNPs with different amounts of AgNPs were synthesized. This protocol was also employed to synthesize the rGO-PtNPs using chloroplatinic acid (H_2_PtCl_6_).

### Preparation of stretchable and conductive rGO-AgNP hybrid films

As-prepared rGO-AgNP hybrid materials (100 mg) were ground with polyvinylidene fluorolide (100 μL of 10 wt% PVDF solution in NMP) using mortar for 30 min and then sonicated for 60 min to make it homogeneously. In the next step, the as-prepared rGO-AgNP ink was printed on to a substrate such as PET or glass using doctor blade technique. And then, the coated rGO-AgNP film was dried (12 hrs under atmosphere condition and 100 °C for 90 min) on a substrate. To prepare a stretchable rGO-AgNP hybrid film, an elastomeric polymer solution was poured on to the as prepared rGO-AgNP film on a substrate and then dried at room temperature for 24 hrs and cured 150 °C for 90 min. After peeled off it from a substrate, the rGO-AgNP hybrid film was also pressed through hot rolling equipment at 150 °C within few seconds. The final dimension of the film was 40 × 5 × 0.03 mm.

### Characterization

Raman spectroscopy measurements were taken using a micro-Raman system (Renishaw, RM1000-In Via) with an excitation energy of 2.41 eV (514 nm). All X-ray photoemission spectroscopy (XPS) measurements were made with a Sigma Probe (ThermoVG, U.K.) with a monochromatic Al-Kα X-ray source at 100 W. The powder X-ray diffraction (XRD) studies were conducted using a D8-Adcance instrument (Germany) and Cu-Ka radiation. The thermal properties of rGO-AgNP materials characterized by TGA (Polymer laboratory, TGA 1000 plus). Microstructure was observed by field emission scanning electron microscopy (SEM, JSM-6701F/INCA Energy, JEOL) and transmission electron microscopy (TEM, JEOL JEM 3010). AFM was performed by using a SPA400 instrument with a SPI-3800 controller (Seiko Instrument Industry Co.) at room temperature. FT-IR spectra were collected using a Thermo Nicolet AVATAR 320 instrument. The conductivities of these hybrid films were measured with a Hall-effect measurement system (HMS-3000, ECOPIA) under varying tensile strain at RT. The stretchabilities of rGO-AgNP hybrid films were carried out using a custom stretching test system.

## Additional Information

**How to cite this article**: Yoon, Y. *et al.* Highly Stretchable and Conductive Silver Nanoparticle Embedded Graphene Flake Electrode Prepared by *In situ* Dual Reduction Reaction. *Sci. Rep.* **5**, 14177; doi: 10.1038/srep14177 (2015).

## Supplementary Material

Supplementary Information
