# Supplementary material for: Highly Stretchable and Conductive Silver Nanoparticle Embedded Graphene Flake Electrode Prepared by In situ Dual Reduction Reaction
Source: Sci Rep. 2015 Sep 18;5:14177. doi: 10.1038/srep14177 (PMC4585658; doi:10.1038/srep14177)
Supplement: Supplementary Information [file srep14177-s1.doc]

**Supplementary Information (SI) for:**

**Highly Stretchable and Conductive Silver Nanoparticle Embedded Graphene Flake Electrode Prepared by *In situ* Dual Reduction Reaction**

Yeoheung Yoon^†1,3^, Khokan Samanta^†2^, Hanleem Lee^2^, Keunsik Lee^2^, Anand P. Tiwari^2^, JiHun Lee^2^, Junghee Yang^2^ and Hyoyoung Lee^1, 2, 3^*

^1^Center for Integrated Nanostructure Physics, Institute for Basic Science (IBS), Sungkyunkwan University, Suwon 440-746. Korea.

^2^Department of Chemistry, Department of Energy Science, SKKU Advanced Institute of Nano Technology (SAINT), Sungkyunkwan University, Suwon 440-746. Korea.

^3^Samsung-SKKU Graphene Center (SSGC), Sungkyunkwan University, 2066 Seoburo, Jangan-Gu, Suwon, Gyeonggi-Do 440-746, Republic of Korea

**.**

**Three dimensional percolation theory**

The electrical conductivity of the rGO-AgNP hybrid films can be described using the power-law relationship as shown in Eq.(1).

σ = σ_0_(*V*_f_ – *V*_c_)*^s^* 1

where σ is the electrical conductivity of the composite,σ_0_ is the electrical conductivity of the filler, *V_f_* is the volumetric fraction of the filler, *V_c_* is the volumetric fraction at percolation threshold and *s* is the fitting exponent. *V*_c_ was calculated using Eq. (2) based on the average inter-particle distance model*.*

*V_c_* = 4(*D + D_IP_*)3/27*πD*^2^*t* 2

where *D* is the diameter of AgNPs, *t* is the thickness of rGO-AgNP hybrid films and *D_IP_* is the distance between conductive fillers for the tunneling of electron (*D_IP =_* 10 nm). *V_c_* = 0.058 was obtained by assuming *D_IP_* = 10 nm leading to an agreement with the experimentally observed percolation threshold (Figure. 5a).The conductivity of bulk silver (σ_0_ = 8.6 x 10^3^ S cm^-1^) was used for Eq. (1), and s=2.47 was obtained from the Chi-Square curve fitting. As shown in Figure. 5a, there was an agreement between the experimental data and theory until the phase separation of Ag flakes was observed.


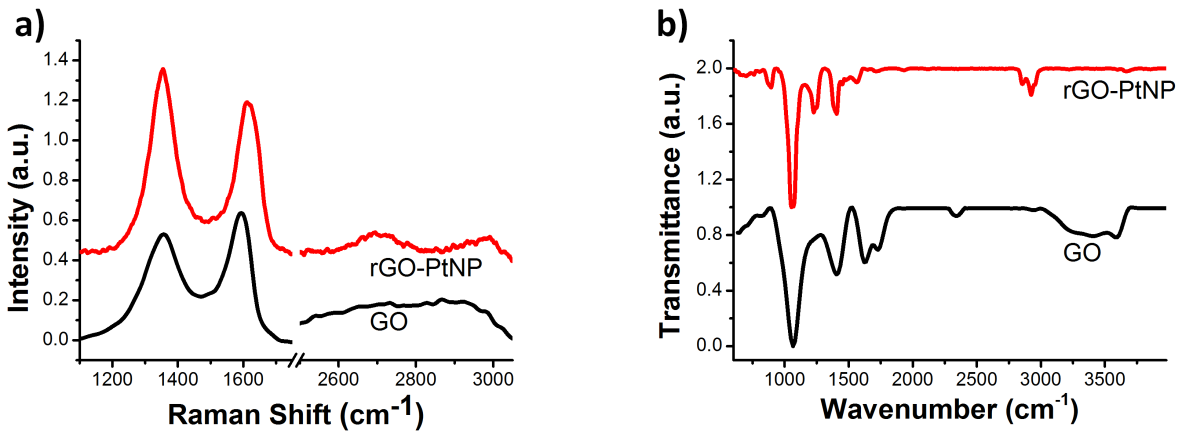


**Figure S1.** Compositional analysis of rGO-PtNP synthesized by using formic acid and hexachloroplatinic acid (H_2_PtCl_6_). (a), Raman spectroscopy of GO and rGO-PtNP hybrid material. (b), FT-IR spectroscopy of GO and rGO-PtNP hybrid material.


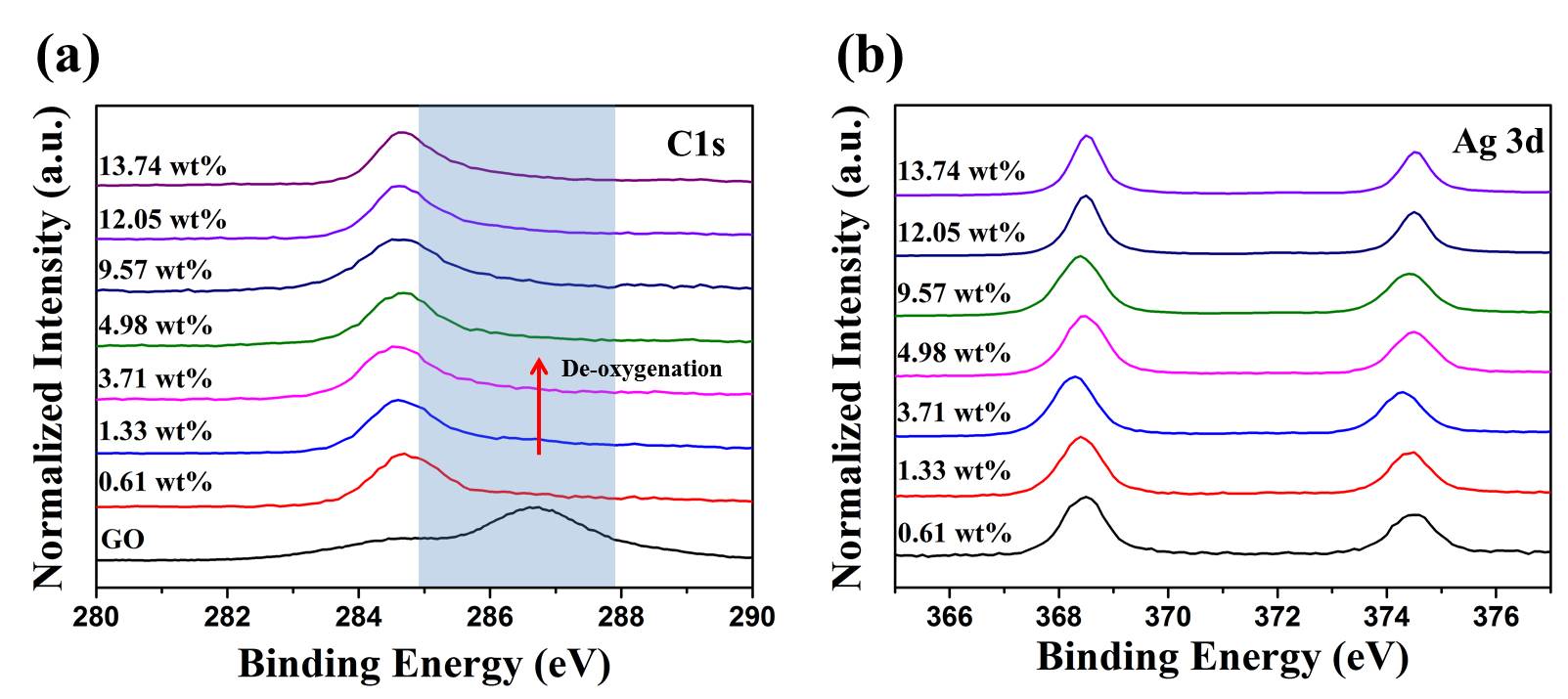


**Figure S2.** High resolution XPS C1s and Ag 3d of the rGO-AgNP hybrid materials having different amount of AgNPs, indicating that the reduction reaction for the preparation of the rGO-AgNP hybrid materials requires a certain standard amount of AgNO_3_ used as an initiator (above 1.33 wt%)


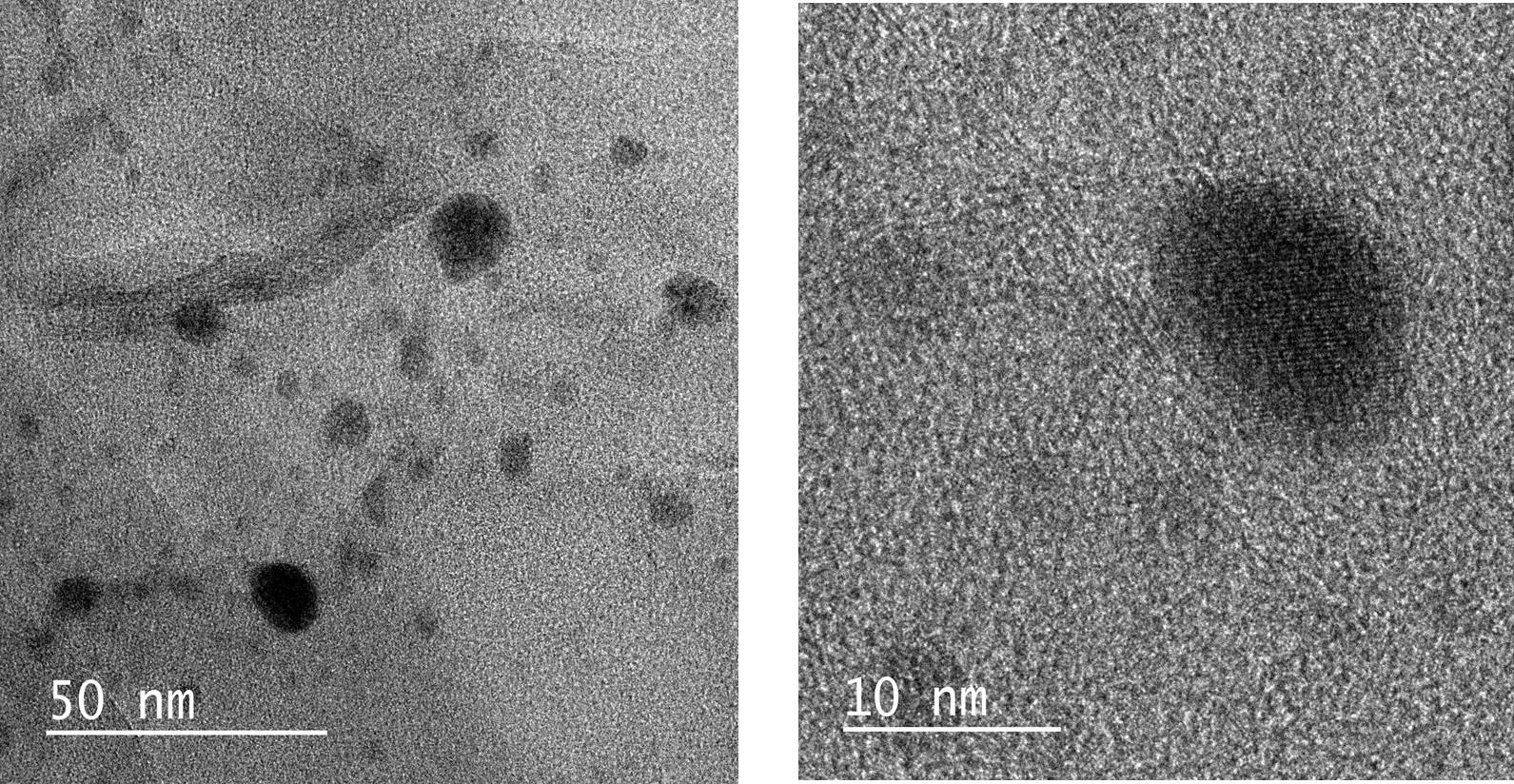


**Figure S3** TEM images of rGO-AgNP hybrid materials


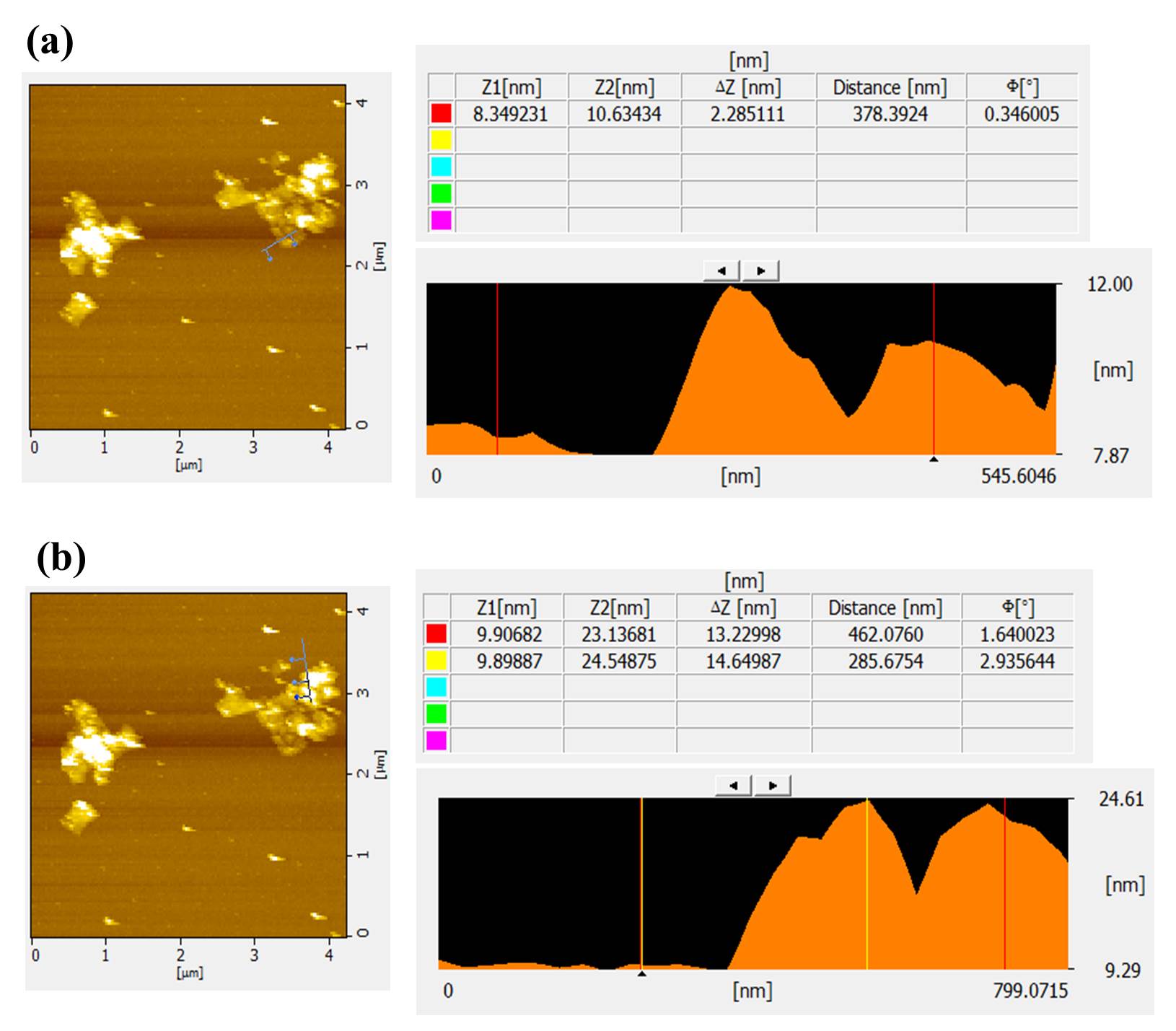


**Figure S4**. AFM images of rGO-AgNPs showing the thickness of the hybrid materials.


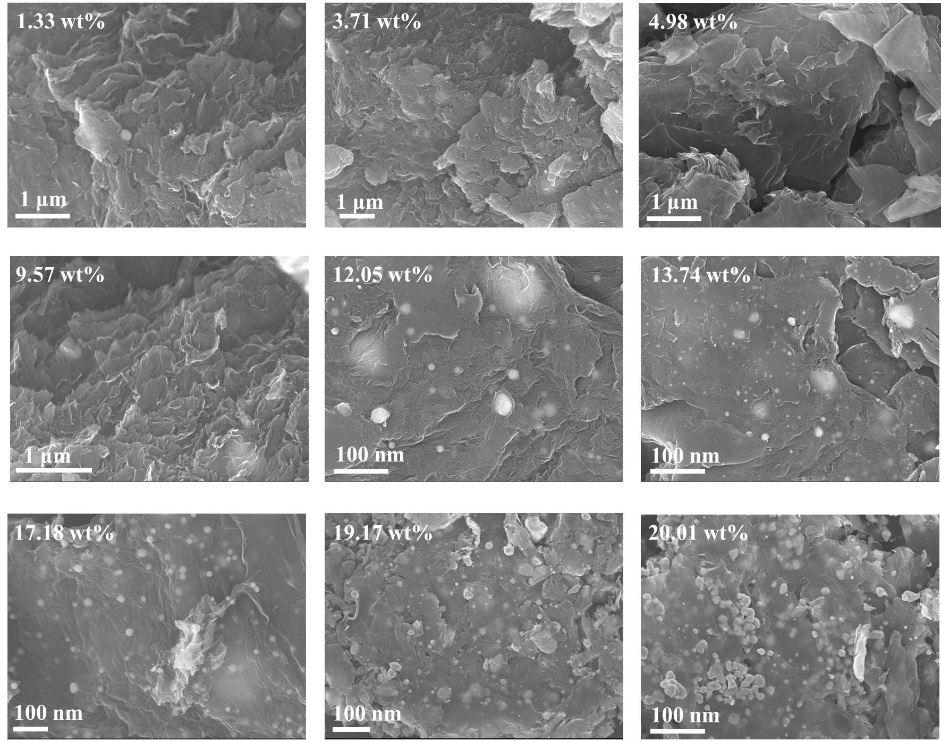


**Figure S5** SEM images of rGO-AgNP hybrid materials having various amount of AgNP


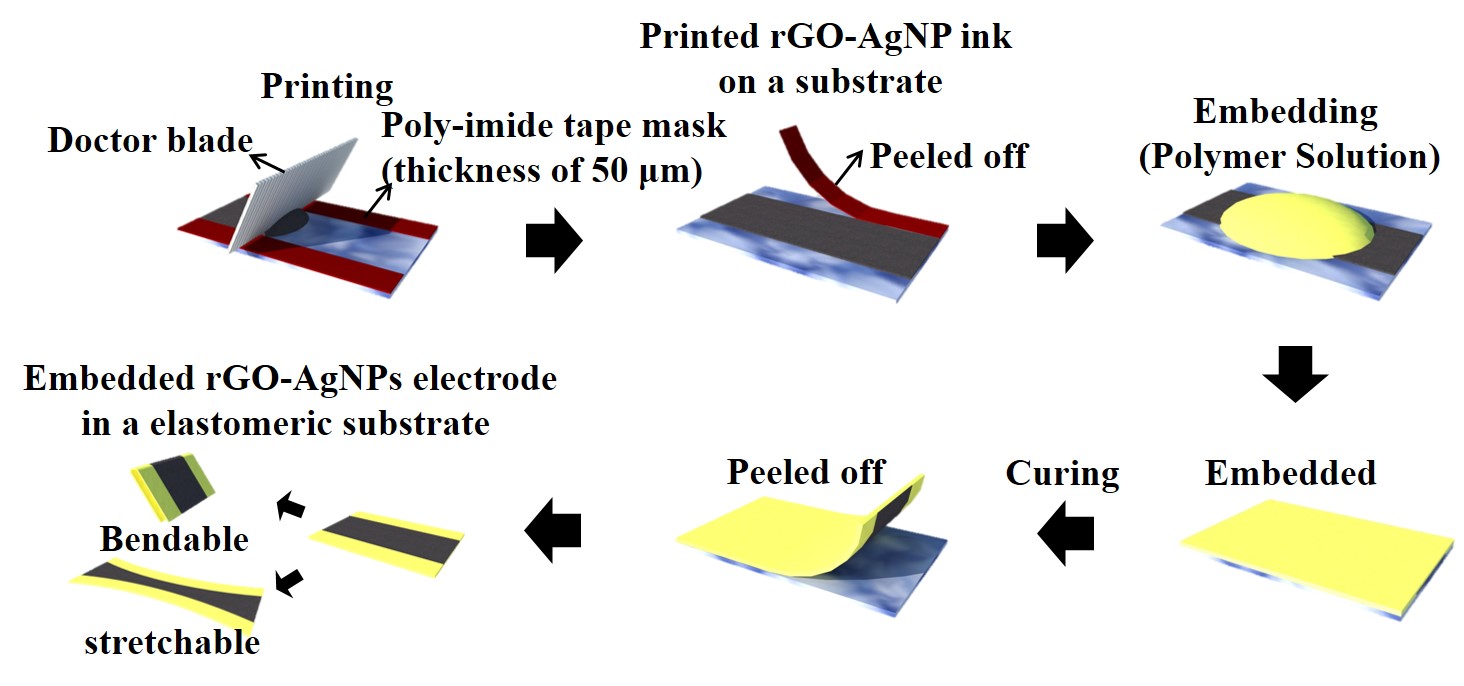


Figure S6 A schematic representation of the fabrication process for the printable, stretchable and conductive rGO-AgNP hybrid films.


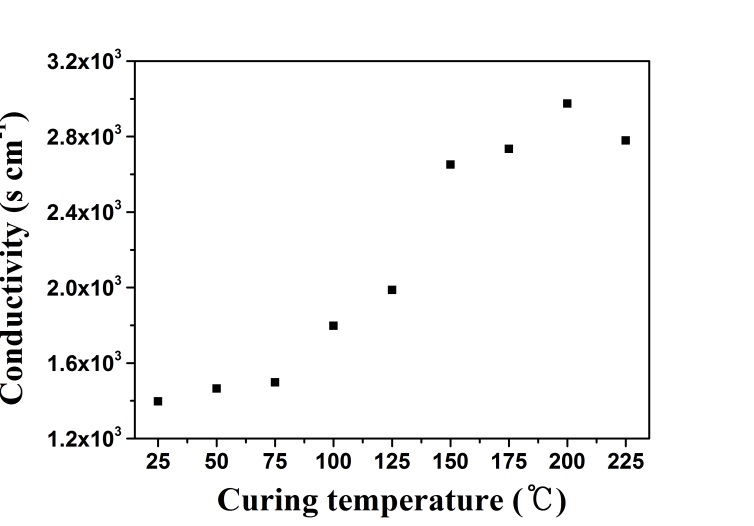


**Figure S7.** Conductivity of the rGO-AgNP hybrid film (Ag atomic percentage of 19.17 wt%) as a function of the Curing temperature.

The conductivity of the rGO-AgNP hybrid film was characterized as a function of the curing temperature. The curing duration was fixed at 90 min. The conductivity was increased as the curing temperature was increased. However, the hybrid film became brittle and the stretchability was significantly reduced above 175 ℃. And also, the conductivity of the hybrid film became rough at above 200 ℃ because a polymer substrate was very weak under high temperature. Therefore, the curing temperature was fixed at 150 ℃ in this research.


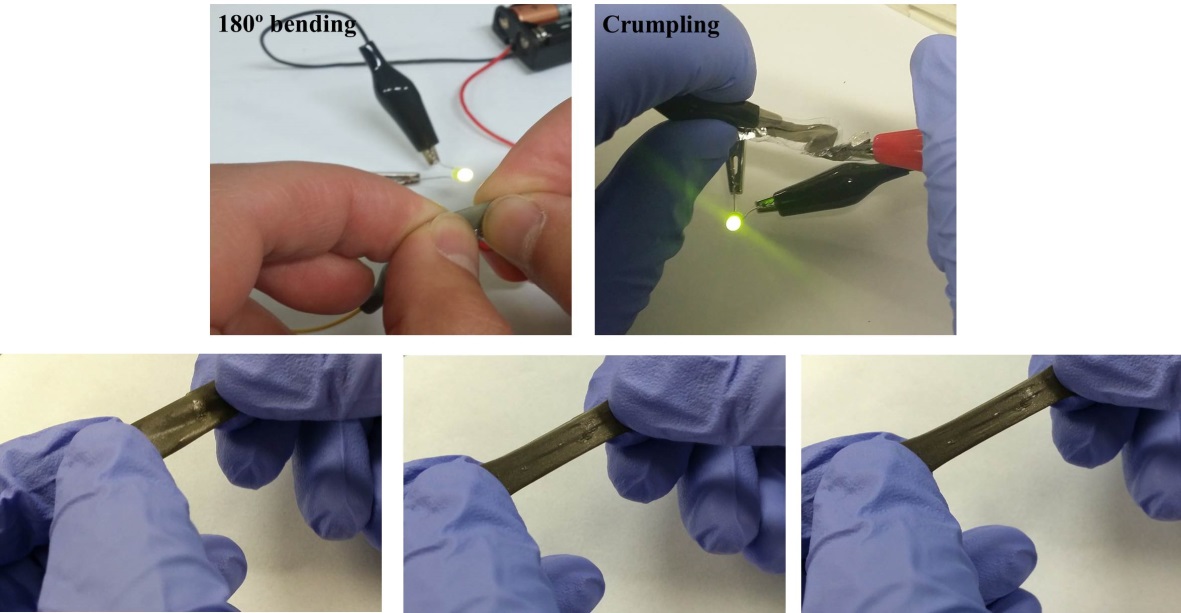


**Figure S8.** Digital pictures of the electrical performance of the rGO-AgNP hybrid film with 180º bending and crumpling test (upper two images). The rGO-AgNP film combined with NBR polymer, which is cured after sonication for 12 hrs, can be stretched up to about 3 times from initial film without any cracking and phase separation (bottom three images).
